# Rational Design Method Based on Techno-Economic Principles for Integration of Organic/Organic Pervaporation with Lipase Catalyzed Transesterification

**DOI:** 10.3390/membranes11060407

**Published:** 2021-05-28

**Authors:** Wouter Van Hecke, Pieterjan Debergh, Mohammed Nazeer Khan, Miet Van Dael

**Affiliations:** 1 Business Unit Separation and Conversion Technology, Flemish Institute for Technological Research (VITO), Boeretang 200, 2400 Mol, Belgium; Pieterjan.Debergh@vito.be (P.D.); mohammednazeer.khan@vito.be (M.N.K.); miet.vandael@vito.be (M.V.D.); 2 Environmental Economics Research Group, Centre for Environmental Sciences (CMK), Hasselt University (UHasselt), Agoralaan, 3590 Diepenbeek, Belgium; 3 Department of Engineering Management, University of Antwerp (UAntwerp), Prinsstraat 13, 2000 Antwerpen, Belgium

**Keywords:** reaction engineering, lipase, pervaporation, transesterification, techno-economic assessment

## Abstract

An engineering foundation is developed in this manuscript to allow the rational design of enzymatic transesterifications integrated with organic–organic pervaporation for the removal of methanol. In the first part, enzyme kinetics are elucidated for the solventless transesterification of two monoterpene alcohols with methyl acetate catalyzed by Novozym 435. Nonlinear regression revealed that three parameters (enzyme loading, forward and backward second-order reaction rate) sufficed to describe the entire conversion as a function of time. In the second part, a mathematical model for acetate ester production, integrated with organic–organic pervaporation, was developed based on a set of ordinary differential equations. To this end, empirical formulae for the pervaporation performance (of a PERVAP 2255-30 membrane and a standard HybSi^®^ membrane) were established, relating methyl acetate and methanol flux to the methanol concentration in the reactor. The resulting digital twin, “PervApp”, allows us to study the influence of the key design parameters “enzyme loading” and “membrane surface” on, e.g., catalyst productivity. Finally, a techno-economic assessment is made for an annual production of 100 tons of geranyl acetate. The described methodology allows producers to shift from laborious, expensive and often disappointing trial-and-error approaches to the rational design of such integrated units.

## 1. Introduction

Lipase catalysis has the edge over conventional catalysis in several cases where sensitive substrates are used [1]. As chemical equilibria are involved in both lipase and conventional catalysis, the completion of (trans)esterification requires an excess of a reagent and/or the continuous removal of byproducts (e.g., by using reactive distillation [2], molecular sieves [1], bubble reactors [3] or pervaporation [4]). A suitable technology for the removal of these byproducts depends on the physicochemical properties of substrates and products. Blowing inert gases through a bubble reactor to remove, e.g., water or methanol may lead to the simultaneous removal of other close-boiling substrates. Continuous reactive distillation was elegantly demonstrated on the pilot-scale for butyl butyrate production in 2017 [5]. Pervaporation is an appealing technology as well, in cases where azeotropic mixtures are involved, and is potentially a less energy consuming technology compared to reactive distillation [6,7]. However, to the best of our knowledge, large-scale industrial applications involving lipase-catalyzed (trans)esterifications integrated with pervaporation have not been implemented so far.

Hydrophilic pervaporation membranes (for water removal) are characterized by outstanding separation factors and fluxes, compared to the separation factor and flux for methanol obtained in organic–organic pervaporation membranes. Separation factors for water of about 10,000 were achieved for feed concentrations of 90% (w/w) ethanol solutions and permeate fluxes of ca. 7 kg/m^2^/h for hydrophilic zeolite membranes [8,9]. Separation factors for methanol have been reported ranging from 7.8 to 3124 [10]. Therefore, if a direct esterification process is technically feasible, but azeotropes with water complicate matters, integration with hydrophilic pervaporation should lead to a more appealing process from a technical point of view. However, water-soluble and dissociable organic acids such as (meth)acrylic and acetic acid (especially when present in high concentrations) severely inhibit and/or inactivate lipases, rendering direct enzymatic esterification for such substrates impractical. This phenomenon was observed for acetic acid as early as 1910 by Bradley [11]. To illustrate this further, increasing the acetic acid concentration from 0.1 to 0.8 M significantly decreased the effectiveness of Novozym 435 [12]. Gubizca et al. [12] reported decreases in reaction rates above 0.3 M acetic acid in n-pentane. Hence, low concentrations of these acids are applied, leading to the introduction of an auxiliary solvent (such as heptane), to low volumetric (and catalyst) productivities due to the kinetics and the high energy expenditure in the downstream processing (to recover the auxiliary solvent and purify relatively low titers of product esters) [13]. The progressive addition of acetic acid is a strategy that has been explored to avoid the negative effect of the organic acid and increase the yield, as reported by Bourg-Garros et al. [14]. In such cases, the acetic acid concentration remains fairly low during the conversion, but this still leads to limited catalyst productivity.

Long-chain fatty acids (such as oleic acid) do not seem to harm the stability of the enzyme [11]. This, in turn, allows the use of solventless conditions, leading to industrially appealing product ester concentrations, high catalyst productivity and a fairly low energy expenditure in the downstream process compared to the diluted systems. Hence, hydrophilic pervaporation technology seems most promising for the direct esterification of long-chain fatty acids with, e.g., ethanol, n-propanol, iso-propanol, n-butanol, iso-butanol, sec-butanol, tert-butanol or allyl alcohol (i.e., compounds that form azeotropes with water).

The use of methyl(meth)acrylate in molar excess for the transesterification of several heavy-boiling alcohols catalyzed by Novozym 435 leads to high product ester titers and near complete conversion of the investigated alcohols (containing aliphatic hydroxyl groups) [1]. In stark contrast to these appealing results, the conversion was insignificant when applying direct esterification using a similar molar excess of (meth)acrylic acid, as applied for transesterification with methyl(meth)acrylates (unpublished results). Therefore, organic–organic pervaporation (for the removal of methanol) is a more promising technology for, e.g., acetate, (meth)acrylate and propionate ester production. In the case of (meth)acrylate ester production, (exothermic) polymerization during transesterification is a risk inherently associated with (meth)acrylates, which has already caused many accidents in chemical industries [15]. Inhibitors such as 4-methoxyphenol (MEHQ) are added to prevent its spontaneous polymerization. The synergy between dissolved oxygen and MEHQ has been described [16] but leads to stringent safety precautions. Anaerobic inhibitors such as phenothiazine (PTZ) can be considered as well, but the rate of inhibitor disappearance has to be known in order to identify the maximum allowable residence time and avoid deleterious events [17]. For technology development purposes, the production of acetate esters is a more prudent approach, as safety issues are greatly minimized.

For both applications (hydrophilic and organic–organic pervaporation) the production price breakdown will be different, as fluxes, separation factors and condensate composition differ. Fluxes mainly depend on permeate pressure, the concentration of water or methanol in the reactor, cross-flow velocity and temperature. These fluxes are seldomly available or published in the literature, let alone in a parameter range encountered during lipase-catalyzed (trans)esterification. The material compatibility of the membranes with the substrates and products of interest is of great importance (e.g., few polymeric materials are compatible with (meth)acrylates). Their long-term stability in pilot or industrial-sized plants is uncertain or publicly unavailable. Although most (industrial) examples relate to hydrophilic pervaporation for water removal [18] (as a stand-alone technology and not in combination with esterification), industrial pervaporation plants have already been commissioned for the separation of methanol from methyl acetate (in 2000) and for the separation of methanol from acetone (in 2002) [19]. Hence, both technologies (hydrophilic and organic–organic pervaporation) have certain desirable features and are reaching industrial maturity.

These technologies also arouse interest for direct integration with (trans)esterification [20,21], but the engineers assigned to design and build such integrated processes currently lack heuristics, design tools or simple rules-of-thumb. This often results in lengthy process development as the design engineer is obliged to apply trial-and-error approaches to gather more insights. As the name of the approach suggests, disappointing or suboptimal results are often obtained in the first rounds of investigation, with the risk of stalling further progress. Engineering tools to robustly predict the process outcome are, to the best of our knowledge, currently unavailable, but would greatly assist in understanding the process and its limitations, in designing and sizing the pervaporation units and in rationally choosing the catalyst loading. The development of “digital twins” is of contemporary interest [22,23,24].

Therefore, in this study, we describe how to shift from a “trial-and-error” approach to a “calculate-and-succeed” approach with minimal experimental effort based on a combination of fundamental (bio)chemical engineering principles (i.e., mechanistic modeling of the enzyme kinetics [25]), combined with empirical modeling to describe pervaporation performance, from a technical and an economic point-of-view.

To this end, enzyme kinetics are elucidated for geranyl and prenyl acetate, both of which are commercially interesting products with floral and fruity aromas used in perfumes and fragrances, air care products, washing and cleaning products, personal care products, etc. Geranyl acetate, for example, is manufactured in (and/or imported to) the European Economic Area at ≥100 to <1000 tons per annum [26].

A forward and backward second-order model is proposed herein to describe the entire progression of the reaction under varying molar ratios of methyl acetate to alcohol and enzyme quantities. Differential equations are established for the integration of transesterification with pervaporation. Finally, the key design parameters, enzyme loading (E) and membrane surface, are varied. Their effect on the residence time required to reach >98% conversion of the alcohol, the productivity of the system and the catalyst productivity of the system are investigated. The result is shown in easy-to-interpret contour plots. The approach is generic and can be applied to other types of esters, where either hydrophilic or organic–organic pervaporation is expected to offer benefits.

Finally, a techno-economic assessment is conducted, using input from the digital twin (PervApp, a portmanteau for Pervaporation Application) with the aim of minimizing production costs by varying key design parameters and with the aim of steering future research efforts based on the cost breakdown. The combination of such digital twins with techno-economic assessments is a powerful approach for steering research efforts and reducing uncertainty in investment decisions prior to building capital-intensive integrated units.

## 2. Materials and Methods

### 2.1. Chemicals

All organic alcohols (geraniol, prenol) and methyl acetate used for the transesterification had purities above 98% and were purchased from Sigma-Aldrich (Schnelldorf, Germany). The desiccant (Molecular Sieve UOP Type 5 Å, Sigma-Aldrich, Schnelldorf, Germany), used to remove methanol, consisted of circular beads with a pore-width of 5 Å. The desiccants were dried for 20 min at 150 °C prior to use.

### 2.2. Analysis

GC-FID (Focus GC, Thermo Fisher Scientific, Waltham, MA, USA) was used to quantify the substrate alcohols and methanol. A FAMEWAX column (30 m × 0.32 mm, with a film thickness of 0.25 µm) was used, obtained from Restek (Middelburg, The Netherlands). The column temperature was initially set to 40 °C and gradually increased to 240 °C. The mobile phase consisted of helium. The injection and detector temperatures were set at 250 °C. Samples were diluted in tetrahydrofuran prior to injection in the GC-FID. At the beginning of each sequence, standards were injected to establish calibration curves. At the end of each sequence, standards were injected for quality control purposes.

The recorded gas chromatograms showed the two substrate peaks, a methanol peak and a fourth peak, identified as the product ester using GC-MS. The product ester concentration (reported as g ester per kg solution, excluding desiccants and catalyst beads) was determined by solving the following mass balance:

(1) $$c_{P}\left[g\cdot {\mathrm{kg}}^{-1}\right]=1000-c_{A}-c_{B}+c_{Q}$$

### 2.3. Reaction Conditions

Both studied alcohols have significantly higher boiling points (230 °C for geraniol; 140 °C for prenol, all at 1 atm) in comparison to methyl acetate (57.1 °C, 1 atm). Therefore, the conversions were only studied in reaction regimes using a molar excess of methyl acetate. This allowed close to complete conversion of the highest-boiling substrate and avoided excessive temperatures in the subsequent distillation tower (to remove the methyl acetate in excess). The conversions were always conducted at 60 °C. Experiments were conducted with desiccants (to remove methanol) and without desiccants (to study the effect of methanol). Initially, the catalyst ratio (E) was varied from 2.5% to 10% during the experiments, similarly to the method used by Heeres et al. for the production of (meth)acrylate esters [1]. However, the change in concentration was so fast that it did not allow a sufficient amount of the sample to be taken in the relevant zones (results are not shown). Therefore, reactions were executed with a decreased E, varying from 0.1 wt% to 2 wt%. The molar ratio of methyl acetate to alcohol was varied from 1.77:1 to 3.38:1.

### 2.4. Calculations

#### 2.4.1. Determination of Mechanistic Model Parameters

In a previous work [1], we successfully proposed a relatively simple second-order model to predict entire conversion profiles (not solely the initial rates), using methyl (meth)acrylate esters as a substrate, along with a series of heavy-boiling alcohols, in the presence of desiccants for the in-situ removal of methanol ($c_{Q}$). This proved to lead to a better prediction of the conversion profile compared to the well-known and often quoted ping-pong reaction mechanism. Therefore, we investigated whether this simple second-order model was generic enough to be expanded to the use of methyl acetate as a substrate, along with heavy-boiling alcohols. In the absence of methanolysis, the reaction rate can be written as:

(2) $$\frac{{\mathrm{dc}}_{P}}{\mathrm{dt}}=k_{1}{\;*\;E\;*\;c}_{A}{\;*\;c}_{B}$$

To determine $k_{1}$, a series of experiments was conducted, in which E and the molar ratio for methyl acetate to alcohol were varied, as described above in Section 2.3.

Furthermore, to confirm previous findings, the suitability of the more commonly applied ping-pong reaction mechanism was investigated by evaluating the following rate equation (for experiments in the presence of desiccants):

(3) $$\frac{{\mathrm{dc}}_{P}}{\mathrm{dt}}=k_{\mathrm{cat}}*E\cdot \frac{c_{A}*c_{B}}{K_{M,A}*c_{B}+K_{M,B}*c_{A}+c_{A}*c_{B}}$$

The differential equations mentioned above fail to predict conversion as a function of time in the absence of desiccants, due to methanolysis and the resulting equilibrium. To predict the course of an integrated pervaporation experiment, the methanol concentration has to be taken into account.

Therefore, the following reaction rate was proposed:

(4) $$\frac{{\mathrm{dc}}_{P}}{\mathrm{dt}}=k_{1}*E*c_{A}*c_{B}-k_{2}*E*c_{P}*c_{Q}$$

This differential equation incorporates the already-introduced forward second-order reaction rate constant $k_{1}$, supplemented with a backward second-order reaction rate constant, $k_{2}$. An equilibrium experiment was conducted to determine $k_{2}$, while fixing $k_{1}$ to the already-determined value. The equilibrium constant is defined as the ratio of the forward reaction to the backward reaction:

(5) $$K_{\mathrm{eq}}=\frac{k_{1}}{k_{2}}=\frac{c_{P}*c_{Q}}{c_{A}*c_{B}}$$

Obviously, $K_{\mathrm{eq}}$ can be determined based solely on the equilibrium concentrations, but to predict the kinetics it is imperative to determine both the forward and backward second-order reaction constant.

All parameter regressions were performed in MATLAB (MathWorks, Natick, MA, USA), using the nonlinear least squares solver function lsqcurvefit with the default ‘trust region reflective’ algorithm. The parameters were regressed from solving a system of ordinary differential equations (for c_A_ and c_B_), using the ode15s solver for stiff differential equations. The developed code used all the experimental data from every conversion simultaneously. The 95% confidence intervals relating to the regressed parameters were calculated using MATLAB based on the Jacobian given by the lsqcurvefit fitting function. 

#### 2.4.2. Pervaporation Experiment

The flux of component i (methanol or methyl acrylate) is measured as:

(6) $$J_{i}=\frac{m_{i}}{S*t}$$

with S signifying the pervaporation surface, t representing the duration of pervaporation and $m_{i}$ signifying the mass of component i.

The separation factor can be expressed as:

(7) αmethanol/methyl acetate=ymethanolymethyl acetatexmethanolxmethyl acetate

with $x_{i}$ signifying the mole fraction of component i in the feed and $y_{i}$ signifying the mole fraction of component i in the permeate.

A ceramic 1-channel membrane tube with a standard Hybrid Silica HybSi^®^ AR top layer was purchased from Pervatech (Rijssen, The Netherlands). The dimensions were 500 mm * 10 mm * 7 mm (L * OD * ID), leading to an exchange surface of 0.011 m^2^ per membrane tube; the membrane (an organic-inorganic hybrid silica-based amorphous material) was coated on the inside [27]. This module was built in an in-house-constructed pervaporation rig, consisting of a temperature-controlled 3-L vessel and a magnetic drive gear pump (Gather, Wülfrath, Germany) to provide a crossflow of 160 L h^−1^ over the pervaporation module, leading to a crossflow velocity of 1.15 m s^−1^. The pervaporation was conducted at 60 °C, which was identical to the temperature of the lipase-catalyzed conversions. The total condensation of permeate vapors was ensured by means of condensation in liquid nitrogen, present in a stainless steel cold trap (KGW-ISOTHERM, Karlsruhe, Germany) prior to the diaphragm vacuum pump (KNF Neuberger GmbH, Freiburg, Germany). The permeate pressure was 5 mbar.

#### 2.4.3. Mathematical Description of Transesterification with Pervaporation

As performance data for pervaporation membranes were hard to find under relevant process conditions, we established an empirical correlation between, on the one hand, the methanol and methyl acetate fluxes and, on the other hand, the methanol concentration in the vessel for the normal Hybsi^®^ membrane (at 5 mbar and 60 °C). Interestingly, methanol and methyl acetate fluxes during pervaporation at 60 °C were published by Gorri et al. [28] for a PERVAP 2255-30 membrane as a function of the methanol concentration. The permeate pressure was kept below 4 mbar. As the original numerical data were not available, we applied Graph Grabber 2.0.2 (Quintessa, UK) to retrieve the relevant data from the published figures.

### 2.5. Techno-Economic Assessment

The main purpose of the TEA is to gain insights into the economic viability of the proposed concept early in its development stage, and to provide guidance for further research activities. By highlighting the main cost factors in the process, research strategies can be designed to alleviate these hurdles. In addition, the TEA can be used to compare different process design options. Of particular interest to the current study is the aim of understanding the impact of the choices with respect to two critical process design parameters, namely, the enzyme loading and membrane surface of the pervaporation unit, on the overall cost.

The TEA was conducted following the general guidelines [29,30]. More concretely, a three-step procedure was followed. First, the mass and energy balances of the process were established. The mass balance of the integrated reaction and pervaporation stage was obtained from the PervApp model described in Section 3.4 and Section 3.5. The energy required to drive the pervaporation through the application of a vacuum was calculated using Aspen Plus V11 Software (Aspentech, Bedford, MA, USA). Similarly, the energy required to separate unreacted methyl acetate from the other components after the reaction (such that it can be recycled) via a distillation procedure was modeled in the same software. Details about these modeling steps can be found in the Supplementary Information (SI), Figures S1 and S2.

Secondly, an economic analysis was directly integrated with the mass and energy balance calculations to assign costs to each step of the process. Information on the cost of the major equipment items (i.e., the bioreactor, pervaporation module, vacuum pump, distillation tower and storage tanks) was collected from the literature and via modeling in Aspen Plus V11, and where necessary converted into 2020 euros using the CEPCI index. Subsequently, multipliers were applied on the equipment cost to account for all other direct and indirect costs that are necessary to build a complete plant. More concretely, equipment-specific multipliers to account for installation costs, as well as multipliers for offsite and contingency costs, were adopted from the work of Towler and Sinnot [31]. The total capital investment obtained via this route was converted into annualized capital investment using a weighted average cost of capital (WACC) of 4.5% and assumptions regarding the economic lifetime of the various equipment items. Operational costs were determined by combining the mass and energy balances with appropriate material and energy prices collected from the literature and supplier information. Personnel costs were determined following the procedure specified by Peters et al. (2003) [32]. All economic assumptions are detailed in the SI, Tables S1–S3. 

As a third and final step, a sensitivity analysis was conducted to understand the impact of the main assumptions on the overall outcome. Considering the large uncertainty that is associated with cost estimation for processes in the early development phase, this is a critical step.

In this study, two main economic metrics, namely, the production cost (PC_GA_) and the conversion cost (CC_GA_), were considered:

$${\mathrm{PC}}_{\mathrm{GA}}=\frac{C_{\mathrm{CAP}}+C_{O\&M}}{{\mathrm{AP}}_{\mathrm{GA}}}$$

with PC_GA_ as the production cost (EUR/kg geranyl acetate), C_CAP_ as the annualized capital investments (EUR/year), C_O&M_ as the operational expenditures (EUR/year) and AP_GA_ as the annual production (kg geranyl acetate/year); and

$${\mathrm{CC}}_{\mathrm{GA}}={\mathrm{PC}}_{\mathrm{GA}}-\frac{C_{\mathrm{GER}\;}+C_{\mathrm{MAc}}+C_{L}}{{\mathrm{AP}}_{\mathrm{GA}}}\;$$

with CC_GA_ as the conversion cost (EUR/kg geranyl acetate), C_GER_ as the geraniol purchase cost (stochiometric requirement) (EUR/year), C_MAc_ as the methyl acetate purchase cost (stochiometric requirement) (EUR/year) and C_L_ as the labor cost (EUR/year).

The production cost is the total cost of manufacturing one kilogram of geranyl acetate. The conversion cost is derived from the production cost, by subtracting the stochiometric required amounts of inputs (geraniol and methyl acetate), as well as labor costs. The main reason for defining this second economic metric is that the conversion cost reflects all costs that can be minimized through improved process design. Hence, this is the main ‘target’ for process innovation. Moreover, production cost values are highly sensitive to assumptions made in regard to input prices (here, in particular, geraniol) and because of the small scale of the process and the amount of labor required, which for processes in the early development stage is usually proxied by a rule-of-thumb only. In practice, production would likely take place in a multi-purpose plant, which would lower the labor cost per unit of product; however, this was left out of the scope of this study. For those reasons, we selected conversion cost as the main metric to be considered here.

## 3. Results and Discussion

### 3.1. Determination of Mechanistic Model Parameters

**Figure 1:**
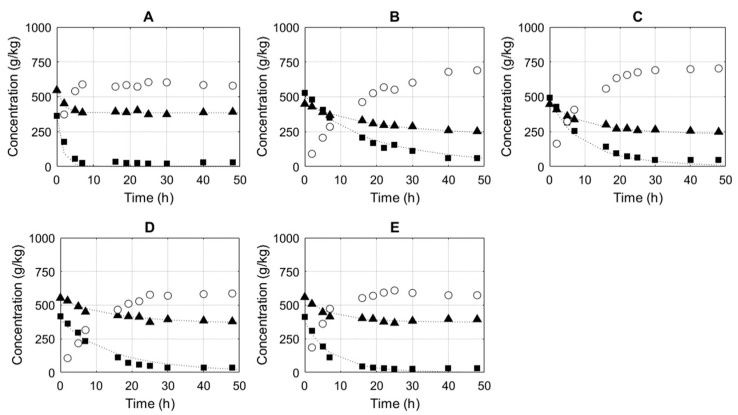
Experimentally determined methyl acetate (▲) and geraniol (■) concentrations and the corresponding second-order model results (…). The experimental geranyl acetate (○) concentration was calculated as described in the Materials and Methods section. E was 1% (**A**), 0.1% (**B**), 0.2% (**C**), 0.1% (**D**) and 0.2% (**E**). The initial molar ratios of methyl acetate to geraniol were 3.11:1 (**A**), 1.77:1 (**B**), 1.88:1 (**C**), 2.78:1 (**D**) and 2.82:1 (**E**).

**Figure 2:**
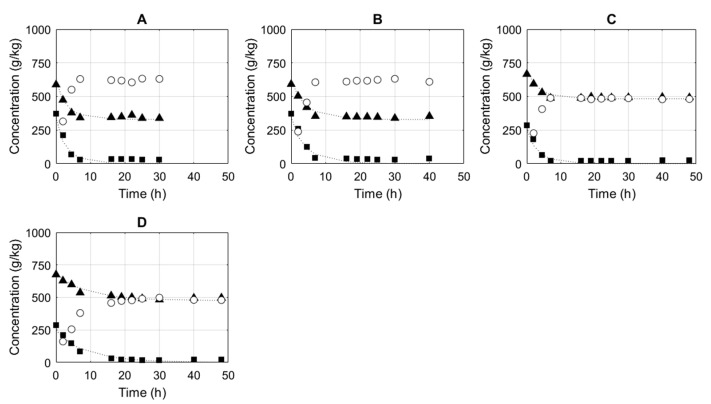
Experimentally determined methyl acetate (▲) and prenol (■) concentrations and the corresponding second-order model results (…). The experimental prenyl acetate (○) concentration was calculated as described in the Materials and Methods section. E was 0.7% (**A**), 0.5% (**B**), 0.5% (**C**) and 0.2% (**D**). The initial molar ratios of methyl acetate to prenol were 1.85:1 (**A**), 1.85:1 (**B**), 2.71:1 (**C**) and 2.76:1 (**D**).

The non-linear regression methodology described by Heeres et al. [1] was used to determine the forward second-order reaction rate constants for the different reactions. The fit with the experimental data is shown in Figure 1 and Figure 2. The confidence intervals (95%) are also provided in Table 1 and the narrowness of this interval further corroborates the suitability of the second-order model to describe the time course of these transesterifications, in the absence of methanol.

The forward second-order reaction constants for methyl acetate are ca. one order of magnitude higher compared to those obtained for methyl (meth)acrylates. Although lipases are known to be promiscuous enzymes with a broad substrate range, methyl acetate is a more favorable substrate in comparison to methyl acrylate and certainly compared to methyl methacrylate. The practical importance of this higher substrate acceptance is underpinned by the significantly lower catalyst quantities required to produce 1 kg acetate esters (in the same time) as 1 kg (meth)acrylate esters. Hence, the cost contribution of Novozyme 435 in the entire production process would be significantly lower for acetate esters in comparison to acrylate, and especially compared to methacrylate esters (for a similar number of cycles).

#### 3.1.1. Geranyl Acetate

Reactions were executed with varying enzyme mass fractions of 0.1 wt% to 1 wt%. The initial molar ratios of methyl acetate to geraniol varied from 1.77:1 to 3.11:1 (E). The experimental data and simulation of the conversion as a function of time are shown in Figure 1. Methanol was not detected throughout the course of the synthesis due to the presence of desiccants.

Regression of the parameters of the ping-pong mechanism resulted in a model curve with a seemingly perfect fit to the experimental data (data not shown). However, the previous findings relating to (meth)acrylate ester synthesis can be expanded to acetate ester synthesis—the regressed parameters failed to have physically meaningful values: k_cat_ = 2310 kg/(mol·h), −11,710–16,331 kg/(mol·h); K_M,A_ = 5.3 (mol/kg), −32.0–42.5; K_M,B_ = 40 mol/kg and −210–289 mol/kg. The 95% confidence interval is extremely large, suggesting that the regressed parameter values are inaccurate and that the lipase does not obey ping-pong kinetics.

On the other hand, the simple second-order model equally has a nearly perfect fit with the methyl acetate and geraniol concentrations (see Figure 1), but the regressed second-order reaction constant has a very narrow confidence interval: k_1_ = 10.3 kg/(mol·h), with 9.61–10.99 kg/(mol·h) being the 95% confidence interval. It can be concluded and confirmed that lipases obey this simple second-order kinetics, not only when using methyl (meth)acrylates, but also when using methyl acetate as a substrate. The methodology succeeds in predicting the entire conversion profile, and not only their initial rates, as frequently encountered in the literature.

**Figure 3:**
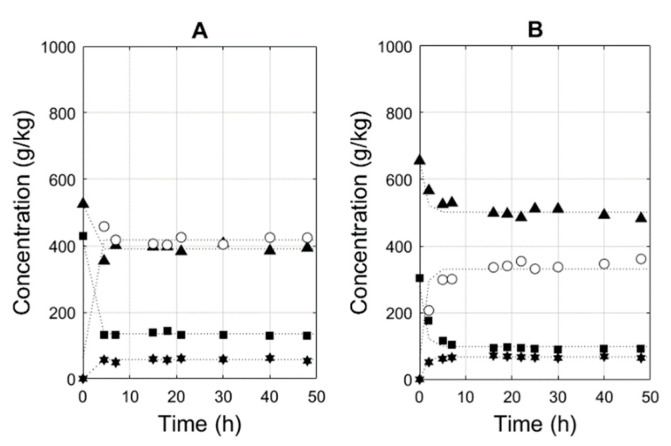
(**A**) Experimentally determined methyl acetate (▲), geraniol (■), methanol (🟌) concentrations and the corresponding second-order model results (…). The experimental geranyl acetate (○) concentration was calculated as described in the Materials and Methods section. E was 2%. The initial molar ratios of methyl acetate to geraniol was 2.55:1. (**B**) Experimentally determined methyl acetate (▲), prenol (■) and methanol (🟌) concentrations and the corresponding second-order model results (…). The experimental prenyl acetate (○) concentration was calculated as described in the Materials and Methods section. E was 1%. The initial molar ratio of methyl acetate to prenol was 2.50:1.

Subsequently, k_2_ can be calculated after the determination of K_eq_ based on an equilibrium experiment (in the absence of desiccants). As an alternative, one can determine k_2_ by means of nonlinear regression of the outcome of an equilibrium experiment by fixing the already-determined k_1_. The result of this fitting procedure can be seen in Figure 3A. The calculated backward second-order reaction constant is 12.45 kg/(mol·h), with 11.01–13.90 kg/(mol·h) as its 95% confidence interval (see Table 2). This is not the first study in which lipases were used to produce geranyl acetate (see Table 3) but to the best of our knowledge, it is the first in which solventless conditions are applied.

#### 3.1.2. Prenyl Acetate

To demonstrate the generic nature of the model, the synthesis of prenyl acetate was studied as well. Reactions were executed with varying enzyme mass fractions of 0.2 wt% to 0.7 wt%. The initial molar ratios of methyl acetate to prenol varied from 1.85:1 to 2.76:1 (E). The experimental data and simulation of the conversion as a function of time and in the presence of desiccants are shown in Figure 2. The experimental results fitted nearly perfectly to the simulation using the simple second-order model. The second-order reaction constant was determined to be 7.29 kg/(mol·h), with 6.35–8.23 kg/(mol·h) as its 95% confidence interval.

Subsequently, the backward second-order reaction constant was calculated to be 10.30 kg/(mol·h), with 8.87–11.73 kg/(mol·h), as its 95% confidence interval (see Table 2). The equilibrium experiment and fit with the model are shown in Figure 3B. Hence, the rate equation mentioned in Equation (4) can be applied to describe the performance of the Novozym 435 for the synthesis of both acetate esters. To this end, only three parameters are required: the catalyst ratio E and a forward and a backward second-order reaction rate. This equation takes into account the presence of methanol and is therefore ideal to use as a rate equation in the generation of a digital twin of the process in which pervaporation is used to remove methanol.

### 3.2. Empirical Model for Pervaporation

Pervaporation is an interesting technology when azeotropes are expected. Energy is mainly consumed to evaporate (and condense) the permeate and to reach and maintain the required vacuum.

We have proven that the conversions can be brought to completion in the presence of molecular sieves. To recover the adsorptive properties, the molecular sieves need to be regenerated by means of pressure swing regeneration (PSR) or temperature swing regeneration (TSR), which require energy. Typically, two columns are built in parallel to allow the regeneration of one column while the other is in operational mode. Apparently, the required size of the packed columns is disadvantageous for this technology [4].

There is no need to have parallel pervaporation units in place, as is the case for absorption columns, to allow full (or semi-)continuous operation. At the end, it is the technology with the lowest production price that will prevail, whether it is through adsorption, reactive distillation, pervaporation or another technology.

**Figure 4:**
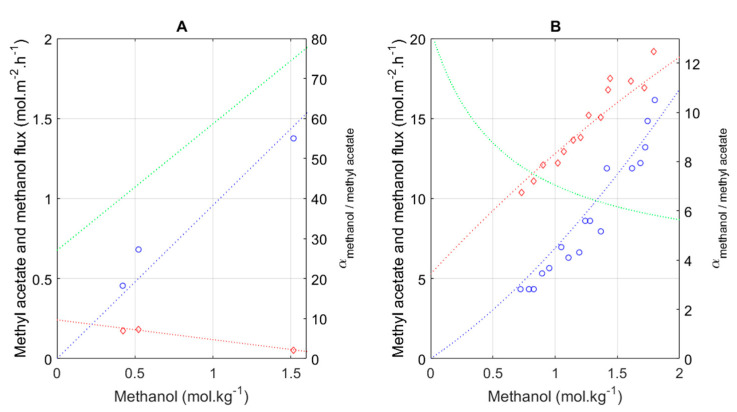
Component fluxes as a function of methanol concentration in the feed for (**A**) standard Hybsi^®^ membrane; (**B**) PERVAP 2255-30 membrane [28]. Symbols: ◇ Methyl acetate flux; ■ Methanol flux. Blue dotted line: simulated methanol flux; red dotted line: simulated methyl acetate flux; green dotted line: simulated separation factor.

We did not find any examples in which enzymatic transesterification was successfully integrated with pervaporation. Integrating two units (reactor + pervaporation) in one hybrid unit requires an intricate knowledge of both technologies. Therefore, the following methodology was developed. The first step is to obtain the performance data of a pervaporation membrane under the relevant conditions. In most cases, these data are hard to find, if they are available at all. The component fluxes as a function of methanol were determined for the normal Hybsi^®^ membrane and are shown in Figure 4A. These results were compared to data from Gorri et al. (2006) [28], who described the performance of a PERVAP 2255-30 membrane (with a PVA-based separating layer and a porous support of PAN) in mixtures of methanol and methyl acetate at 60 °C. The methanol and methyl acetate flux can be seen in Figure 4B. Although total fluxes were significantly lower for the standard Hybsi^®^ membrane in comparison to the PERVAP 2255-30 membrane, the separation factor was significantly higher.

The following empirical equations were used to describe the methyl acetate flux $J_{B}$ and methanol flux $J_{Q}$ for the PERVAP 2255-30 and standard Hybsi^®^ membrane:

(8) $$J_{B\_\mathrm{PERVAP}}=0.027*{\left(c_{Q}\right)}^{3}-0.82*{\left(c_{Q}\right)}^{2}+8.3*(c_{Q})+5.3$$

(9) $$J_{Q\_\mathrm{PERVAP}}=-0.072*{\left(c_{Q}\right)}^{3}+1.72*{\left(c_{Q}\right)}^{2}+5.25*(c_{Q})$$

(10) $$J_{B\_\mathrm{HYBSI}}=-0.1233*(c_{Q})+0.2421$$

(11) $$J_{Q\_\mathrm{HYBSI}}=0.9576*(c_{Q})$$

In a second step, a model was developed, integrating the pervaporation with transesterification.

### 3.3. Model Development Integrating Transesterification with Pervaporation

A variable reactor content is taken into account in these equations. Only methyl acetate and methanol can be removed by pervaporation; the others lack volatility. The initial mass of the reactor is represented as $m_{R,0},$ whereas the mass of the reactor at time t is represented as $m_{R}$. The simplify the mathematics, the reaction rate equations were transformed into molar flow rates ($\dot{m}$). Through substitution of Equation (4), the following molar flow rates describe the transesterification:

(12) $${\dot{m}}_{A}={\dot{m}}_{B}=-{\dot{m}}_{P}=-{\dot{m}}_{Q}=E\;*\;\frac{m_{R,0}}{m_{R}^{2}}*\left(-k_{1}*n_{A}*n_{B}+k_{2}*n_{P}*n_{Q}\right)$$

The infinitesimal changes in reactor weight, molar flows of A, B, P and Q, can then be described as:

(13) $$\frac{{\mathrm{dm}}_{R}}{\mathrm{dt}}=-S*(J_{B}*{\mathrm{MW}}_{B}+J_{Q}*{\mathrm{MW}}_{Q})/1000$$

(14) $$\frac{{\mathrm{dn}}_{A,R}}{\mathrm{dt}}={\dot{m}}_{A}$$

(15) $$\frac{{\mathrm{dn}}_{B,R}}{\mathrm{dt}}={\dot{m}}_{B}-S*J_{B}$$

(16) $$\frac{{\mathrm{dn}}_{P,R}}{\mathrm{dt}}={\dot{m}}_{P}$$

(17) $$\frac{{\mathrm{dn}}_{Q,R}}{\mathrm{dt}}={\dot{m}}_{Q}-S*J_{Q}$$

The infinitesimal changes in condensate weight of the condensate can be described as:

(18) $$\frac{{\mathrm{dn}}_{B,C}}{\mathrm{dt}}=S*J_{B}$$

(19) $$\frac{{\mathrm{dn}}_{Q,C}}{\mathrm{dt}}=S*J_{Q}$$

(20) $$\frac{{\mathrm{dm}}_{C}}{\mathrm{dt}}=-\frac{{\mathrm{dm}}_{R}}{\mathrm{dt}}$$

### 3.4. Simulations of Conversion as a Function of Time

Simulations with the PERVAP 2255-30 membrane led to disappointing results, despite their high fluxes. Due to the low separation factor, the (simulated) large methyl acetate removal impeded a full conversion of geraniol for all molar ratios (up to an initial excess of 5 moles of methyl acetate to 1 mole of geraniol). Even if the use of this membrane would have led to a desirable outcome, the relatively low separation factor leads to excessive condensation energy costs. Even though the results are disappointing, the digital twin proves its merit—without performing a single (experimental) integrated experiment, the PERVAP 2255-30 membrane can be discarded, saving resources for more promising membranes. Potentially, the PERVAP™4155 membrane can be considered for follow-up experiments as it is marketed as being particularly suitable for the removal of methanol from volatile components [36], but to the best of our knowledge detailed performance data are not available. Therefore, the following simulations were performed with the Hybsi^®^ membranes which were characterized by low(er) fluxes but higher separation factors in comparison to the PERVAP 2255-30 membranes.

**Figure 5:**
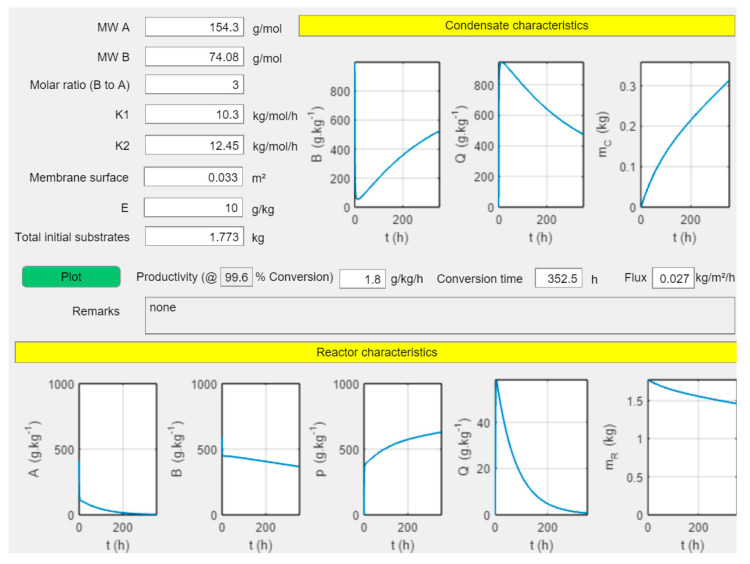
Screenshot of “PervApp” for geranyl acetate production with the progression of the reaction as a function of time with input parameters mentioned above-left.

In the digital twin “PervApp” the condensate composition, condensate mass (m_C_), reactor composition and reactor mass (m_R_) were all modeled. Figure 5 shows a screenshot of the PervApp for the production of geranyl acetate. The molar ratio (methyl acetate to geraniol) is three, the membrane surface 0.033 m^2^ and the ratio of catalyst mass to initial substrate is 10 g kg^−1^. This leads to a productivity of just 1.8 g kg^−1^ h^−1^ and an excessive conversion time of 352.4 h (>14 days), at which point 99.6% of the alcohol is converted. The specific membrane surface in this case is 18.6 m^2^/1000 kg (initial substrate).

**Figure 6:**
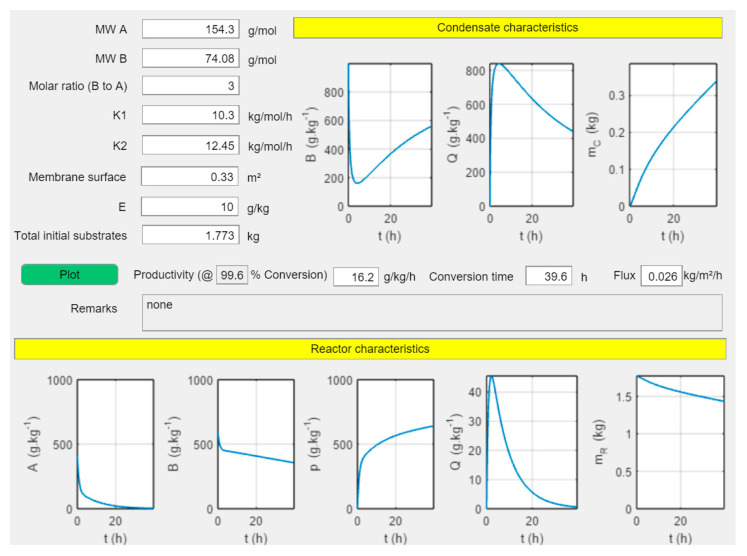
Screenshot of “PervApp” with the progression of the reaction as a function of time with input parameters mentioned above-left. The condensate composition, condensate mass (m_C_), reactor composition and reactor mass (m_R_) are all plotted.

Increasing the catalyst loading “E” does not shorten the conversion time. The progression of the reaction is hampered by the membrane area. Hence, increasing the surface leads to shorter conversion times. In Figure 6, only the membrane surface has been increased to 0.33 m^2^. This leads to a productivity of 16.2 g kg^−1^ h^−1^ and a conversion time of 39.6 h. The specific membrane surface in this case is 186 m^2^/1000 kg (initial substrate). The conversion time can be decreased further by increasing the catalyst loading. A catalyst loading of 28 g kg^−1^ leads to a conversion time of 36 h, with all other parameters as before. Hence, an instrument to evaluate the effect of key design parameters (enzyme loading and membrane area) on the progression of the reaction as a function of time is at our disposal. 

### 3.5. Effect of Membrane Surface and Enzyme Ratio

**Figure 7:**
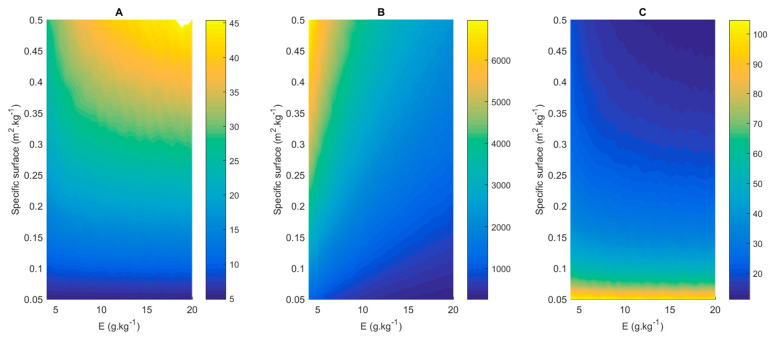
Contour plots to illustrate the effect of specific membrane surface (m^2^ membrane per kg initial substrate) and enzyme loading on (**A**) productivity (in mol/kg/h), (**B**) specific productivity (mol/h/kg_catalyst_) and (**C**) the conversion time (h) required to reach >98% conversion of the (heavy-boiling) alcohol. A molar ratio of 4 (methyl acetate to geraniol) was applied in this simulation.

To further explore the effect of the key design parameters of enzyme loading and membrane surface, contour plots were created (see Figure 7), where productivity, specific productivity and the conversion time required to reach >98% conversion were plotted as a function of enzyme loading (x-axis) and specific surface (y-axis). In these plots, the interaction between many process-related parameters becomes clear. 

**Figure 8:**
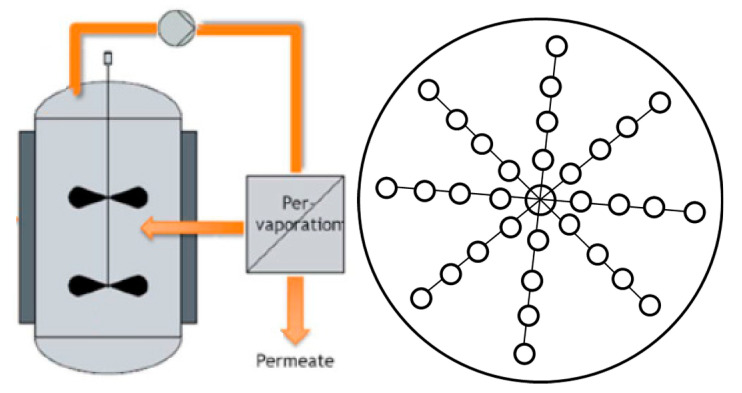
**Left**: conventional set-up, consisting of reactor vessel, crossflow pump and pervaporation module(s); **right**: top view of a reactor equipped with dynamic pervaporation (DynaPer).

However, these results pose new questions. For this particular membrane, it is clear that a large specific area is required to decrease the conversion time. In conventional set-ups (Figure 8 left), a crossflow pump recirculates liquid from a temperature-controlled vessel to the pervaporation membranes and back. If the membrane area needs to be enlarged, either more modules in series are installed (inherently leading to concentration and temperature gradients which are not taken into account in the model development) or they are installed in parallel (leading to a higher crossflow to keep the crossflow velocity constant). Interstage heaters are frequently applied to counter temperature gradients. To avoid concentration and temperature gradients and to avoid high crossflow demands, a (patented) design was developed that avoids concentration and temperature gradients along the length of the membranes. The dynamic pervaporation (DynaPer) concept consists of pervaporation membranes (and filtration membranes to allow continuous production), mounted on an oscillating rotor (Figure 8, right). Other envisioned advantages are increased safety, as potentially toxic chemicals are not recirculated at high flow rates, and the lower footprint of such an installation compared to a conventional set-up [37]. We believe this process intensification strategy could be utilized for reactors from 20 L to 1 m^3^, but this should be investigated further.

Obviously, the flux can be increased by increasing the temperature further. Currently, tests have been performed for the standard Hybsi^®^ membrane at 60 °C, but this particular membrane can handle temperatures up to 150 °C, where fluxes are much higher. The optimal temperature for the Novozyme 435 seems to be 40–60 °C. Presumably, the operating temperature can be increased further, leading to higher second-order reaction constants (according to Arrhenius’ law) but also to higher inactivation rates. This trade-off could be explored further. It could equally be an option to explore this membrane further in combination with, e.g., Amberlyst catalysts, where recommended operational temperatures between 120 °C and 170 °C can be found [38]. In such cases, the permeate pressure could be higher as well, leading to lower energy requirements for condensation.

The long-term stability of membranes for methanol removal is an unknown factor, as most studies report very limited durations for this experiment [39]. Once a membrane is selected and characterized, long-term tests should be performed to check whether the membrane performance deteriorates as a function of time.

### 3.6. Techno-Economic Assessment

Finally, the most important question is which combination of membrane surface, reactor size and enzyme loading will lead to the minimum production price. A techno-economic assessment was performed for 100 ton per annum of geranyl acetate production. The influence of membrane surface and enzyme loading were investigated using PervApp as the input.

**Figure 9:**
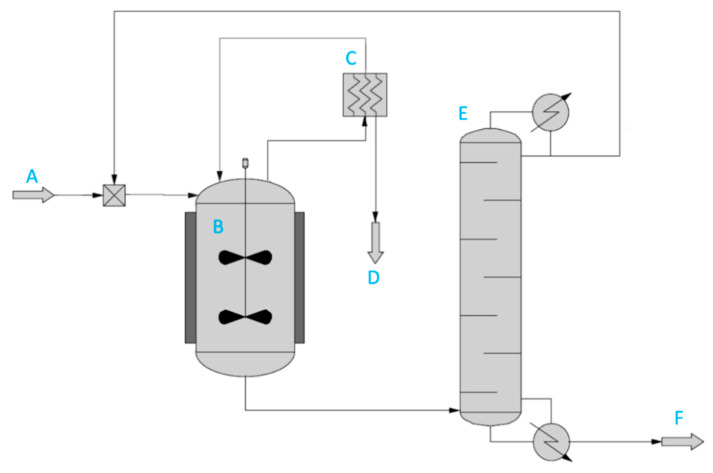
Simplified process flow diagram for the integration of transesterification with pervaporation, including recovery and recycle loop. (**A**) Methyl acetate and alcohol feed; (**B**) reactor; (**C**) pervaporation unit; (**D**) permeate; (**E**) distillation column, where top stream with unconverted methyl acetate is recycled to reactor; (**F**) product ester.

The model developed above solely describes the integration of pervaporation with transesterification. The overall process (Figure 9) is a reactor-separator-recycle system [40], involving a distillation column to separate the product ester (in the bottom) from the methyl acetate added in excess (present in the top stream). The enzyme is either suspended in the reactor, in cases where a lyophilized enzyme is used, or packed in a column (not drawn), in the case where it is immobilized to avoid mechanical damage to the beads. In cases where high-boiling alcohols are used, it is recommendable to fully convert these in the reactor because these are harder to separate in the distillation column from the product ester. However, in cases where low-boiling alcohols are used as substrates, the transesterification should not necessarily be driven to completion, as higher productivities can be reached at lower conversion yields, saving reactor costs. The residual (low-boiling) substrate alcohol can be separated from the product ester in the distillation tower, along with the residual methyl acetate. The recycled substrate(s) from the top stream can then be recycled in the process. Ultimately, it is the overall process performance and economics that count. In this manuscript, the first case (heavy boiling alcohols as substrate) is elaborated, wherein we strive for a near-complete conversion of the high-boiling substrate alcohol in the reactor. The pervaporation permeate contains a mixture of methanol and methyl acetate. Methyl acetate cannot be completely removed from methanol through distillation, due to the existence of the azeotrope. In the TEA, this mixture was considered as a byproduct. Such mixtures are used as solvents for paint or spirit-based coatings [41]. It could equally be separated by extractive distillation [42], but this was not considered in this manuscript.

**Figure 10:**
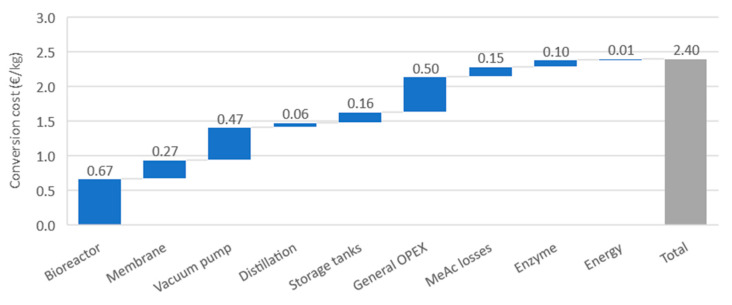
Breakdown of conversion costs for the baseline scenario.

Based on the economic assumptions provided in the SI, Tables S1–S3, and the reaction data provided by the PervApp model, the conversion cost was calculated for the baseline scenario. This baseline scenario represents the combination of specific membrane surface (m^2^/kg initial substrate) and enzyme loading (g/kg initial substrate), for which the lowest conversion cost was found (the procedure through which this optimum was found is elaborated below). Figure 10 provides a cost breakdown. On the equipment side, the cost of the bioreactor, pervaporation module and vacuum pump are the most important. It should be kept in mind that in practice, given the small production volumes of geranyl acetate, production would likely be carried out in a multi-purpose plant where multiple esters are made. Because of scale effects, capital costs per unit of product would be lower for the multi-purpose plant than for the one-product plant simulated here.

On the side of operational costs, the methyl acetate fraction that is lost via permeation and the enzyme consumption are the most relevant cost factors. Insurance, repair and maintenance costs, grouped under the term ‘general OPEX’, are large contributors to operational costs as well. However, as these are calculated as an annual fixed percentage of capital investments, they in fact indirectly reflect the height of the latter. Steam and electricity costs, grouped together under the term ‘energy’, have only a minor contribution to the overall cost.

This graph provides valuable insights in understanding how improved system performance could reduce costs. In particular, a membrane with higher fluxes and better selectivity would be beneficial. The higher fluxes would reduce reaction times, lowering the amount of membrane required and reducing bioreactor costs as batch size decreases. A more selective membrane would reduce the costs of methyl acetate losses, as well as the capital cost of the vacuum pump, as less material would need to be removed through pervaporation.

A breakdown of production costs shows that the geraniol input price and labor costs constitute the main cost factors, with a share of 52% and 38%, respectively (SI, Figure S3). The share of the conversion cost is around 10% in the optimized case. Production cost levels in the range of 22–23 EUR/kg ester are found under the assumptions made, which seem competitive in relation to the market price of 25 EUR/kg that we identified [43]. However, care should be taken with this comparison because, as noted earlier, in practice production would be carried out in a larger, multi-purpose plant, with different economics.

**Figure 11:**
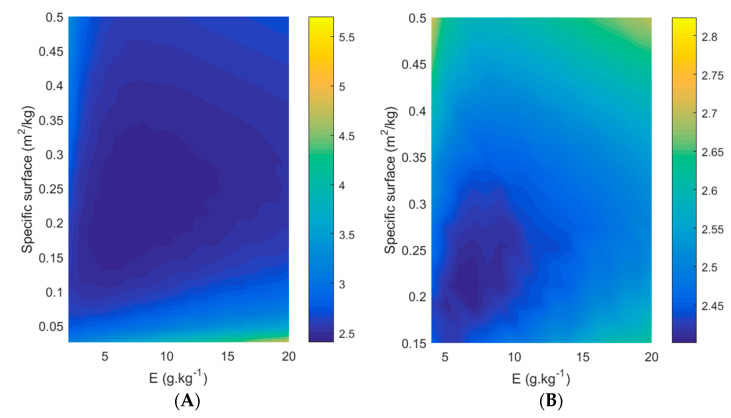
Contour plot of conversion costs as a function of pervaporation membrane surface (m^2^/kg substrate) and enzyme loading (g/kg substrate). Blue reflects lower than average costs, yellow reflects higher than average costs. Conversion costs (in EUR/kg) are indicated in color bars to the right of the contour plot. (**A**) Specific surface ranges from 0.03 m^2^/kg to 0.5 m^2^/kg while enzyme loading ranges from 2 to 20 g kg^−1^; (**B**) Specific surface ranges from 0.15 m^2^/kg to 0.5 m^2^/kg while enzyme loading ranges from 4 to 20 g kg^−1^.

Figure 11 shows contour plots similar to those shown in Section 3.5, with enzyme loading and membrane surface on the horizontal and vertical axes, respectively; however, this Figure displays conversion cost values (in EUR/kg). The information depicted in Figure 11A and Figure 11B is identical, but the range of the y-axis is different, to enable the easy observation of the economic optimum. The availability of the PervApp model, providing reaction times required to reach >98% conversion of geraniol for all possible combinations of enzyme loading and membrane surface, allows us to search across these combinations for an economic optimum. Based on the contour plots, a number of observations can be made. Firstly, a clear optimum can be identified (in subplot B: enzyme loading 7 g kg^−1^, specific surface 0.191 m^2^ kg^−1^), with contour lines of increasing conversion cost around it. Secondly, steeply increasing costs at the bottom of subplot A can be observed, which represent cases with low specific membrane surfaces and, as a result, rather long reaction times (>100 h). Thirdly, for a given amount of enzyme loading, the conversion cost first decreases as membrane surface is increased; however, after a certain point the cost increases again. The difference between the highest and the lowest values found in the plots is more than a factor two, and even reaches a factor of five when considering values which have not been plotted for readability reasons, indicating that this optimization exercise is highly relevant.

**Figure 12:**
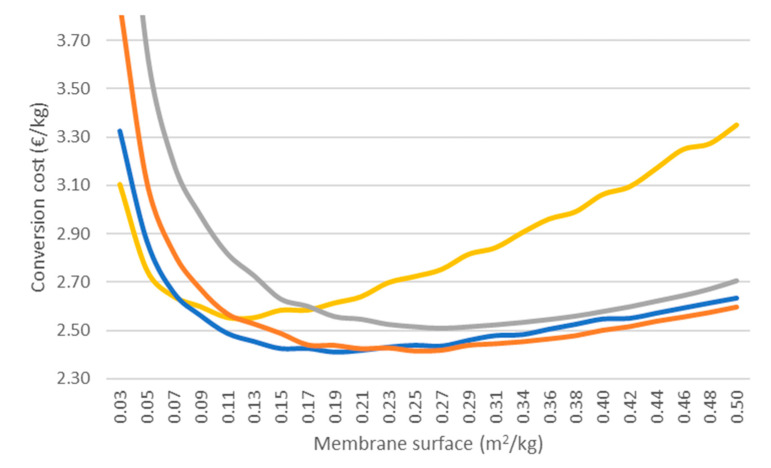
Conversion cost as a function of pervaporation membrane surface and enzyme loading. 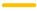 E = 2 g kg^−1^; 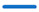 E = 5 g kg^−1^; 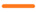 E = 10 g kg^−1^; 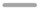 E = 20 g kg^−1^.

To shed more light on these observations, Figure 12 plots conversion cost as a function of specific membrane surface for four different enzyme loadings (i.e., vertical cross-sections of the contour plot). For each enzyme loading, a clear economic optimum can be identified, namely, the specific membrane surface corresponding with the lowest point on the curve. The position of this optimum depends on the enzyme loading: the higher the enzyme loading, the higher the corresponding optimal specific membrane surface. We can also observe high costs in the case of low enzyme loading (2 g/kg) at high specific membrane surfaces (the top-right part of the figure), and conversely, for high enzyme loadings at low specific membrane surfaces (the top-left part of the figure). Clearly, this suggests the existence of an optimal ratio between these two parameters. In cases of high enzyme loadings combined with low specific membrane surface, the reaction is presumably constrained by the removal of methanol. Adding more enzyme does not lead to significantly shorter reaction times, but only to higher costs. In the case of high specific membrane surfaces combined with low enzyme loadings, the reaction is presumably constrained by the lack of catalytic activity. Adding more membrane surface only leads to additional costs. The increased removal of one of the inputs, methyl acetate, may also play a role in this case.

At the same time, it can also be observed that across a rather broad range of specific membrane surface values (roughly speaking, the middle part of Figure 12) and enzyme loadings (5 to 10 g/kg), conversion costs are not vastly different. This implies that from a design and engineering point of view, a safety factor in enzyme loading can be applied to take into account inactivation, without significant repercussions on conversion costs. Once below the threshold of 0.05–0.07 m^2^/kg, conversion costs start increasing significantly for all enzyme loadings considered in the analysis, as reaction times increase to well above 100 h per batch. Such long reaction times impact the business case in a very negative way.

In Table 4, more detail on the interaction between reaction characteristics and process economics is provided. We compare three scenarios: one with an optimal ratio between specific membrane surfaces and enzymes (‘Baseline’), one in which the enzyme loading is increased strongly (‘High enzyme’), and one in which the membrane surface is reduced strongly (‘Low surface’). When comparing the first two scenarios, we see that adding a high amount of enzymes reduces the conversion time only in a limited way (~12%). Despite this, capital cost remains at the same level, as the slightly lower reactor cost is cancelled out by higher enzyme column costs. Moreover, the extra enzyme consumption leads to a significantly higher conversion cost. When comparing the first and third scenarios, we see that lowering the specific membrane surface by a factor of 10 has a dramatic effect on conversion time. Because of this, capital costs increase as batch size increases. Interestingly, the enzyme costs also increase as more enzyme is required in absolute terms. In addition, even though the specific membrane surface is 10 times lower, membrane costs decrease only by ~25% due to the larger required net reactor volume, and more membrane surface is required in absolute terms. This highlights the importance of realizing sufficiently short reaction times.

It should be kept in mind that the economic optima described above depend on the assumptions made. For example, an increase in enzyme price will shift the optimum towards lower enzyme loadings.

**Figure 13:**
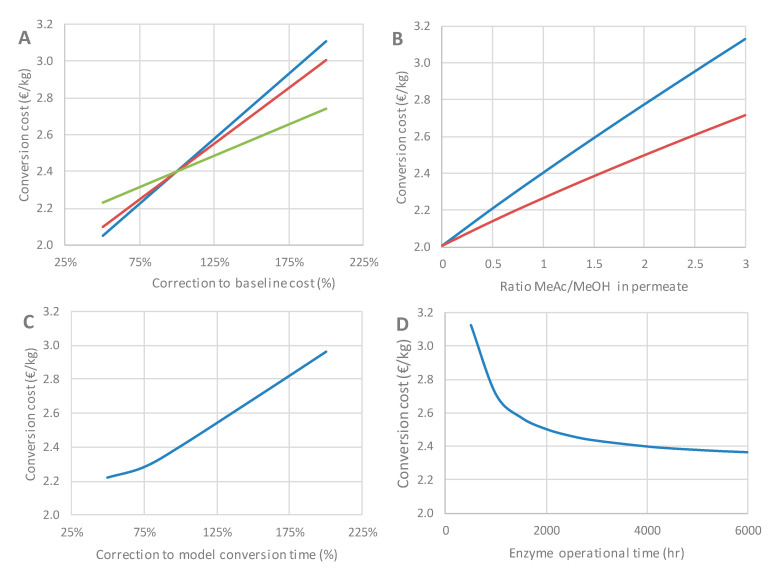
Impact of assumptions regarding capital investments (**A**), permeate composition (**B**), conversion time (**C**) and enzyme operational time (**D**) on conversion cost. Plot (**A**): 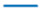 Reactor; 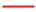 Vacuum pump; 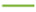 Membrane module. Plot (**B**): 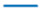 no methyl acetate recovery; 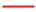 full methyl acetate recovery.

A sensitivity analysis allows us to evaluate the impact of changes in these assumptions and identify the parameters with the highest impact on the calculated production and conversion cost. The sensitivity of the conversion cost to six key parameters is shown in Figure 13. Bioreactor, membrane and vacuum pump costs were selected for sensitivity analysis as these represent the main capital investments (see Figure 10). Considering the inherent uncertainty of CAPEX estimates for processes that are still at the laboratory scale, it is important to take into account the fact that the final cost of the plant could deviate substantially from the baseline value. As can be observed, the highest sensitivity was found for bioreactor cost, as doubling the baseline cost (normalized at 100%) yields a larger increase than for the membrane module and vacuum pump (Figure 13A). The cost of the bioreactor is also one of the main differentiators between cases with short and longer reaction times (the latter requiring larger batch sizes). Different assumptions on the degree of cost increases associated with reactor size increases (i.e., scale effects) can alter the evaluation of the economic feasibility of cases with longer reaction times.

The baseline assumption for membrane module costs is 300 EUR/m^2^, based on literature data. Uncertainty about this parameter can be thought of as reflecting the purchase price of the membrane, as well the uncertainty in the costs of engineering and upscaling the high specific membrane surface. Interestingly, the impact of doubling or halving the membrane cost is fairly limited, in the order of 10%–15% of the conversion cost.

Regarding the technical parameters, conversion time and methyl acetate losses (both simulated via the PervApp model), as well as enzyme reuse (average operational time), were considered for sensitivity analysis.

According to the PervApp model, the composition of the permeate would in most cases (including the baseline scenario) be around 50% methyl acetate and 50% methanol, but in specific cases higher shares of methyl acetate can also be obtained (70%–80%). Clearly, this represents a potential economic loss, as this methyl acetate fraction is not used as intended. Although the investigation of procedures to recycle this methyl acetate fraction was left out of the scope of this study, it should be noted that methyl acetate and methanol constitute an azeotropic mixture [28], separating these two components is not straightforward. As shown in Figure 13B, the difference between the best-case (no methyl acetate loss) and worst-case scenarios (high loss of methyl acetate via permeation) is large. The full recovery of methyl acetate would alleviate, but not fully avoid, these higher costs, since a high share of methyl acetate in the permeate also requires a higher vacuum pump capacity and therefore leads to additional costs.

The impact of reaction time on the conversion cost is evaluated by introducing a parameter, which is used to artificially correct the reaction times provided by the PervApp model by a certain percentage (100% = baseline). This sensitivity analysis can be thought of as reflecting the uncertainty in the modeling of the integrated transesterification–pervaporation system. Doubling the conversion time yields a cost increase of about 25% (Figure 13C).

Regarding the average enzyme operational time, the baseline assumption is 4000 h. Deviating from this assumption does not seem to have a great impact on the cost, as long as the average operational time is above 1000 h (Figure 13D). Below this threshold, costs increase significantly. This element is to be investigated in the future, as lab-scale experiments are generally not performed over such elevated time spans. The impact of other parameters, such as reactor temperature, on enzyme activity should also be considered.

It should be stressed that the sensitivity analysis presented here applies only to the baseline (cost-optimal) scenario. For cases with different values for specific membrane surfaces and enzyme loadings (and hence, a different cost structure), the impact of a change in a certain parameter may be very different.

Finally, the analysis presented above shows the power of combining reaction engineering with process economics. This analysis allows for the design of an integrated unit for transesterifications combined with pervaporation with limited experimental input. Information from relatively simple tests (enzyme and membrane characterization) allows the design engineer to estimate the process parameters and costs of a production plant. The modeling shows us that the optimal selection of the specific membrane surface and enzyme loading is required for overall cost minimization. Hence, the model can be used as a direction-steering tool to further increase the technology readiness level in regard to transesterification combined with pervaporation.

## 4. Conclusions

A mathematical model based on ordinary differential equations was developed to describe the kinetics of a transesterification process integrated with pervaporation. The enzymatic reaction was simulated with the use of a mechanistic second-order model. Three parameters were sufficient to describe enzyme catalysis: (1) the forward second-order reaction, (2) the backward second-order reaction and (3) the lipase loading. Empirical equations were established to predict pervaporation performance. Subsequently, contour plots were created based on 1500 simulations to visualize the effects of key design parameters, i.e., lipase loading (in g enzyme per kg initial substrate) and membrane surface (in m^2^) on productivity, required residence time and catalyst productivity (per batch). We are convinced that this approach will greatly assist design engineers in shifting from laborious and expensive trial-and error approaches to the rational design of such integrated units based on solid engineering principles. Techno-economic assessment allowed us to define an optimal combination of reactor size, membrane surface and catalyst loading, leading to minimal production costs.

## 5. Patents

Van Hecke, W., Elslander, H., Vanbroekhoven, K., De Wever, H., Beckers, H. EP3679150A1. 2020. Method and apparatus for in situ product recovery. 

## Figures and Tables

**Table 1:** Regressed forward second-order reaction rate constants, ranked in decreasing order. The lower and upper limit (LL and UL) of the 95% confidence intervals (CI) are also given.

Component A	Component B	k_1_ kg/(mol·h)	CI LL kg/(mol·h)	CI UL kg/(mol·h)	Source
Geraniol	Methyl acetate	10.3	9.61	10.99	This study
Prenol	Methyl acetate	7.29	6.35	8.23	This study
Tetrahydrofurfuryl alcohol	Methyl acrylate	1.69	1.26	1.71	[1]
Citronellol	Methyl acrylate	1.31	1.18	1.45	[1]
Tetrahydrofurfuryl alcohol	Methyl methacrylate	0.35	0.31	0.38	[1]

**Table 2:** Regressed backward second-order reaction rate constants, ranked in decreasing order. The lower and upper limit (LL and UL) of the 95% confidence intervals (CI) are also given.

Component A	Component B	k_2_ kg/(mol·h)	CI LL kg/(mol·h)	CI UL kg/(mol·h)	Source
Geraniol	Methyl acetate	12.45	11.01	13.90	This study
Prenol	Methyl acetate	10.30	8.87	11.73	This study

**Table 3:** Enzymatic synthesis of geranyl acetate.

Study	Geraniol	Conversion	Lipase	Solvent
Claon and Akoh (1994) [33]	0.12 M	Up to 99%	*C. antarctica* lipases (SP382 and SP435)	n-hexane
Yee and Akoh (1996) [34]	0.1 M	Up to 97%	*Pseudomonas* sp. lipase	n-hexane
Molinari et al. (1998) [13]	0.11 M	55%	Dry mycelium of *Rhizopus delemar* MIM	n-heptane
Peres et al. (2003) [35]	0.2 M	100%	Novozym 435	sc. Ethane and CO_2_
This study	3.2 mol/kg	>98%	Novozym 435	solventless

**Table 4:** Comparison of reaction characteristics and costs for three different scenarios.

Parameter	Unit	Baseline	High Enzyme	Low Surface
Enzyme loading	g/kg initial substrate	7	30	7
Membrane surface	m^2^/kg initial substrate	0.191	0.191	0.026
Conversion time	h	26	23	163
Number of batches	#	279	307	51
Geraniol (input)	ton/year	79	79	79
Methyl acetate (input)	ton/year	151	151	151
Novozyme	ton/year	0.01	0.04	0.07
Reactor size	m^3^	0.90	0.82	4.90
Membrane area	m^2^	157	143	118
Ratio membrane size/reactor size	m^2^/m^3^	175	175	24
Geranyl acetate (output)	ton/year	100	100	100
Enzyme costs	EUR/year	10,356	39,363	65,640
Membrane cost (annualized)	EUR/year	22,614	20,553	16,958
Total CAPEX cost (annualized)	EUR/year	163,599	165,068	207,924
Conversion cost	EUR/kg ester	2.4	2.7	3.6

## Data Availability

The data presented in this study are available in Supplementary Information.

## Supplementary Materials

The following are available online at https://www.mdpi.com/article/10.3390/membranes11060407/s1. Table S1: Overview of equipment cost assumptions; Table S2: Overview of assumptions for the calculation of total capital cost; Table S3: Operational expenditure assumptions; Table S4: Stream conditions in a vacuum pump (see Figure S1); Table S5: Stream conditions in the distillation column; Figure S1: Model representation of a vacuum pump; Figure S2: Model representation of a distillation column; Figure S3: Breakdown of production cost in the baseline scenario; Figure S4: Sensitivity of production cost to the assumption regarding geraniol price in the baseline scenario; Figure S5: Sensitivity of production cost to the assumption regarding the amount of operating labor in the baseline scenario.

## Abbreviations

AP_GA_	Annual production geranyl acetate (kg year^−1^)
$c_{A}$	Alcohol concentration (mol kg^−1^)
$c_{B}$	Methyl acetate concentration (mol kg^−1^)
C_CAP_	Annualized capital investments (EUR year^−1^)
C_GER_	Geraniol purchase cost (EUR year^−1^)
C_L_	Labor cost (EUR year^−1^)
C_MAC_	Methyl acetate purchase cost (EUR year^−1^)
C_O&M_	Operational expenditures (EUR year^−1^)
$c_{P}$	Product ester concentration (mol kg^−1^)
$c_{Q}$	Methanol concentration (mol kg^−1^)
CC_GA_	Conversion cost geranyl acetate (EUR kg^−1^)
E	Ratio of catalyst mass to total substrate mass (g g^−1^)
GC-FID	gas chromatography-flame ionization detector
GC-MS	gas chromatography-mass spectrometry
GUI	graphical user interface
ID	Inner diameter (mm)
$J_{B}$	Methyl acetate flux (mol m^−2^ h^−1^)
$J_{Q}$	Methanol flux (mol m^−2^ h^−1^)
$k_{1}$	Forward second-order reaction constant (kg mol^−1^ h^−1^)
$k_{2}$	Backward second-order reaction constant (kg mol^−1^ h^−1^)
$k_{\mathrm{cat}}$	Specific rate constant (kg mol^−1^ h^−1^)
$K_{M,A}$	Michaelis–Menten constant for alcohol (mol kg^−1^)
$K_{M,B}$	Michaelis–Menten constant for methyl acetate (mol kg^−1^)
L	Length (mm)
LL	Lower limit of the 95% confidence interval for the specific rate constant (kg mol^−1^ h^−1^)
${\dot{m}}_{}$	molar flow rate (mol h^−1^)
MEHQ	4-methoxyphenol
MW	Molecular weight (g mol^−1^)
OD	Outer diameter (mm)
PC_GA_	Production cost, geranyl acetate (EUR kg^−1^)
PVA	polyvinyl alcohol
PTZ	phenothiazine
r	Productivity (g kg^−1^ h^−1^)
S	Exchange surface of the pervaporation membrane (m^2^)
s.c.	supercritical
UL	Upper limit of the 95% confidence interval for the specific rate constant (kg mol^−1^ h^−1^)
$x_{i}$	mole fraction of component i in the feed
$y_{i}$	mole fraction of component i in the permeate
