# Supplementary material for: Rational Design Method Based on Techno-Economic Principles for Integration of Organic/Organic Pervaporation with Lipase Catalyzed Transesterification
Source: Membranes (Basel). 2021 May 28;11(6):407. doi: 10.3390/membranes11060407 (PMC8229130; doi:10.3390/membranes11060407)
Supplement: Supplementary file 1 [file membranes-11-00407-s001.zip › membranes-1222500-supplementary.pdf]

membranes

Supplementary Information: Rational Design Method Based on Techno-Economic Principles for Integration of Organic/Organic Pervaporation With Lipase Catalyzed Transesterification

Wouter Van Hecke, Pieterjan Debergh, Mohammed Nazeer Khan and Miet Van Dael

Economic assumptions

Capital investment

Table S1. Overview of equipment cost assumptions.

Item	Cost	Source	Notes
Reactor	C= 12,800 + 88,200 * $V^{0.4}$<br>With C= reactor cost ($2010); V= reactor volume (m^3)	[1]	
Membrane module	C=30 * A<br>With C= module cost ($2001), A= membrane surface (ft^2)	[2]	
Vacuum pump	C= 2,200,000 €<br>Applies to a permeate removal capacity of 97 kg/h at 5 mbar	Modelling in Aspen Plus V11	A scaling factor of 0.8 was used to estimate cost at lower scale
Enzyme column	C=0.057 * $(V/1000)^{0.3169}$ * 1,000,000<br>With: C= column cost (€2019), V= column volume (L)	[2]	Included in the article under the category 'reactor'
Recirculation pump	C=EXP(9.2951 – 0.6019 * LN(S) + 0.0519 * $(LN(S))^2$)<br>With: C= pump cost ($2001), S = flow rate ((gal/min) * $ft^{0.5}$)	[2]	Included in the article under the category 'reactor'
Storage tanks	C= 210 * $V^{0.51}$<br>With: C= tank cost ($2001), V= tank size (gallons)	[2]	
Distillation unit	C= 64,000 €<br>Applies to a capacity of 484 kg/h	Modelling in Aspen Plus V11	A scaling factor of 0.6 was used to estimate cost at lower scale

Total capital investments was derived from equipment cost via the following formula:

$$\mathrm{TCI} = \left(\sum_{i} \mathrm{EC_i} * \mathrm{IF_i}\right) * (1 + \mathrm{OS}) * (1 + \mathrm{CO}) \quad (1)$$

Where:
TCI= total capital investment (€)
EC_i= Equipment cost for item *i* (€)
IF_i= installation factor for item *i*
OS= multiplier for offsite costs (%)
CO= multiplier for contingencies (%)
The values of these respective multipliers are provided in Table S2

Table S2. Overview of assumptions for the calculation of Total Capital Cost.

Multiplier	Item	Value	Source
IF_i	Reactor	4	[1]
	Membrane module	4	Own assessment
	Vacuum pump	2.5	[1]
	Enzyme column	2.5	Own assessment
	Recirculation pump	4	[1])
	Storage tanks	2.5	[1]
	Distillation unit	4	[1]
OS	Whole plant	30%	[1]
CO	Whole plant	10%	[1]

Operational expenditures

Table S3. Operational expenditure assumptions.

Type	Amount	Unit	Source
Labor—operating	2	# shifts	[3]
Labor—management & supervision	25	% of operating labor cost	[1]
Wage cost	40	€/h	Eurostat (country: Belgium)
Repair	1	% of TCI/year	[1]
Maintenenace	2	% of TCI/year	[1]
Insurance	0.5	% of TCI/year	Industry sources
Electricity price	93	€/MWh	Eurostat (for medium scale industrial consumer in Belgium)
Steam	26	€/ton	Aspen
Enzyme	1000	€/kg	Vendor information
Geraniol	15	€/kg	ICIS
Methyl acetate	850	€/ton	ECHEMI

Aspen models

The vacuum pump was modeled using compressors in three intercooled stages. The permeate from the pervaporation step was a mixture of methyl acetate and methanol (60:40 wt.%) at 60 °C and vacuum pressures. The permeate was compressed from the vacuum pressure to atmospheric pressure in three stages with constant pressure ratio. The permeate after each compression stage was cooled down to 30 °C in order to save compression energy. The utility used for intercooling was cooling water at 18 °C heated until 30 °C. The pressure loss during intercooling was assumed as 1% of the inlet pressure. At the end of the vacuum pump, a condenser was introduced to condense the permeate to atmospheric temperature (25 °C). Figure S1 and Table S4 shows the model representation of the vacuum pump and the stream conditions, respectively. Distillation was performed at atmospheric pressure by using a RadFrac unit operation in Aspen plus. The molar reflux ratio specified was 0.1 and the boil-up ratio was adjusted to 1.56 in order to achieve 97% recovery of methyl acetate with traces of other components. The geranyl acetate stream obtained from the reboiler was 97% pure. The distillation model representation and the stream conditions are shown in Figure S2 and Table S5, respectively.

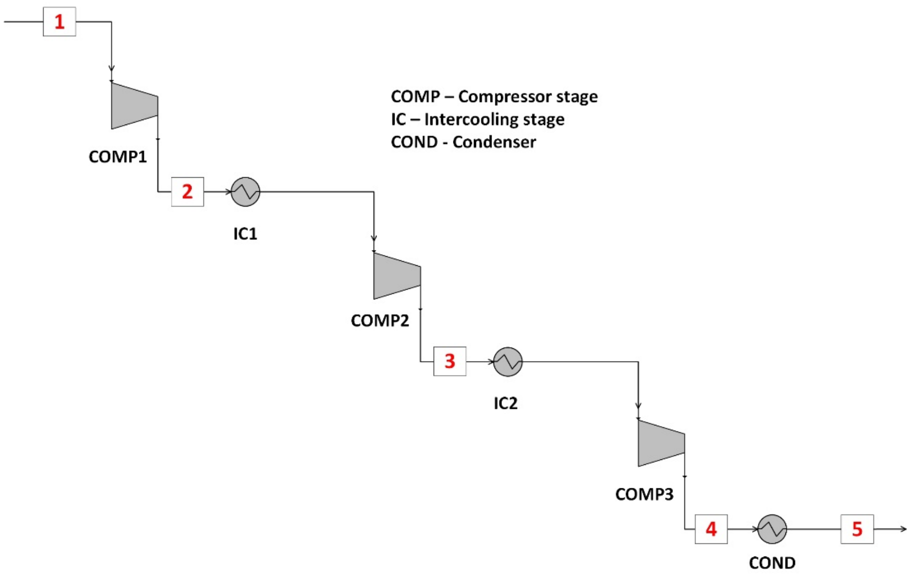

Figure S1. Model representation of a vacuum pump.

Table S4. Stream conditions in a vacuum pump (See Figure S1).

Stream	Temp.	Pressure	Mass flow	Mole fractions (%)	
	(°C)	(bar)	(kg/s)	Methyl acetate	Methanol
1	60	0.005	96.5	39.4	60.6
2	152.7	0.03			
3	119.9	0.174			
4	120.6	1.023			
5	25	1.013			

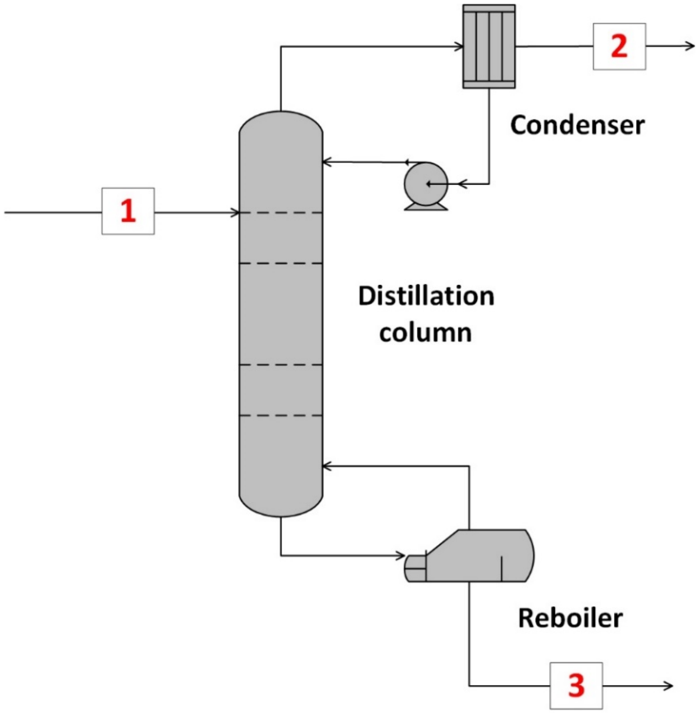

Figure S2. Model representation of a distillation column.

Table S5. Stream conditions in the distillation column

Stream	Temp. (°C)	Pressure (bar)	Flow (kg/s)	Mole fractions (%)		
				Methyl acetate	Geranyl acetate	Geraniol
1	60	1	484.6	73.8	26.1	0.1
2	57	1	247.5	100	0	0
3	195.5	1	237	2.7	97	0.3

Production cost breakdown

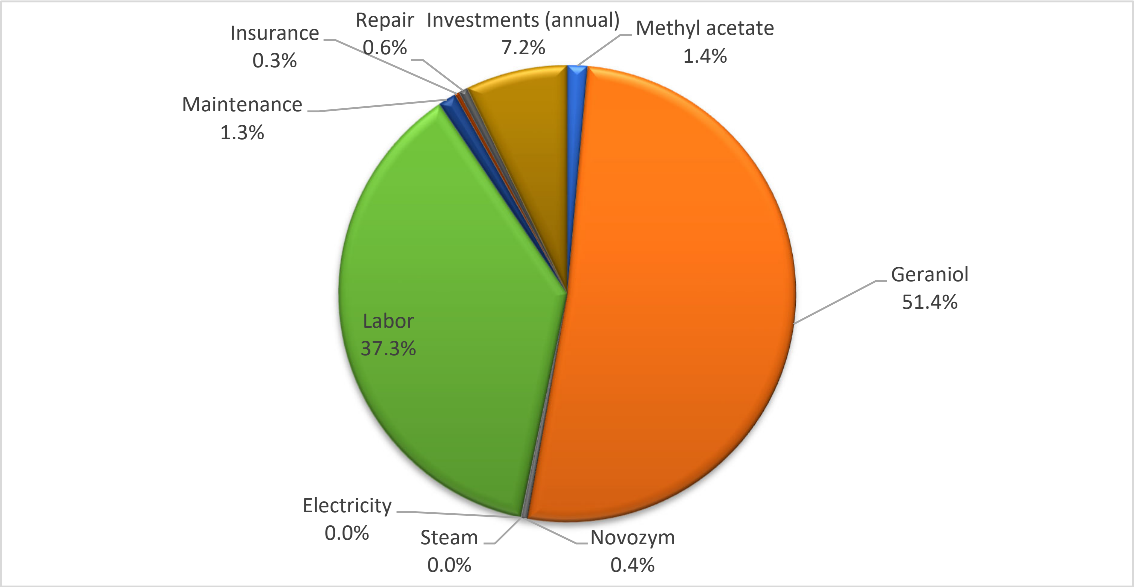

Figure S3. Breakdown of production cost in the baseline scenario.

Sensitivity analysis

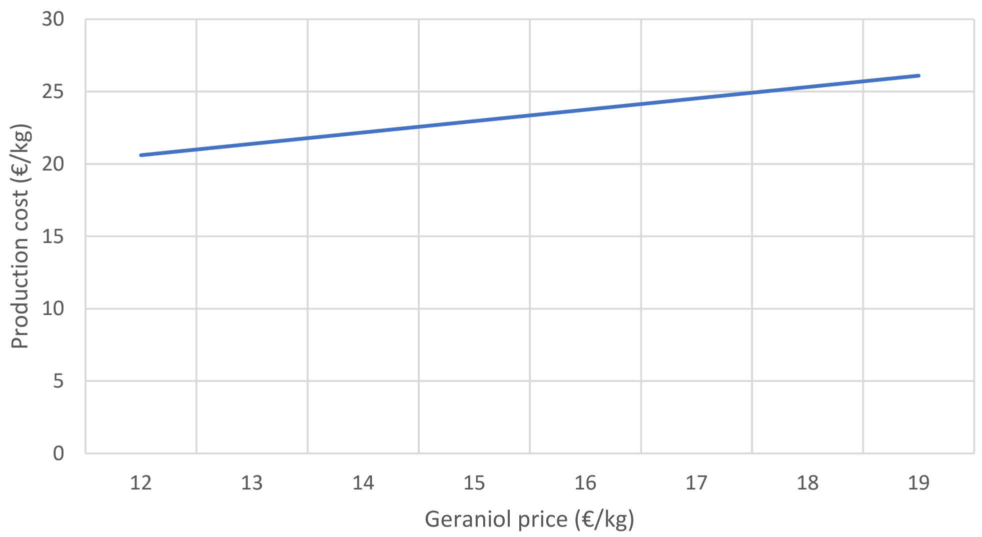

Figure S4. Sensitivity of production cost to the assumption on geraniol price in the baseline scenario.

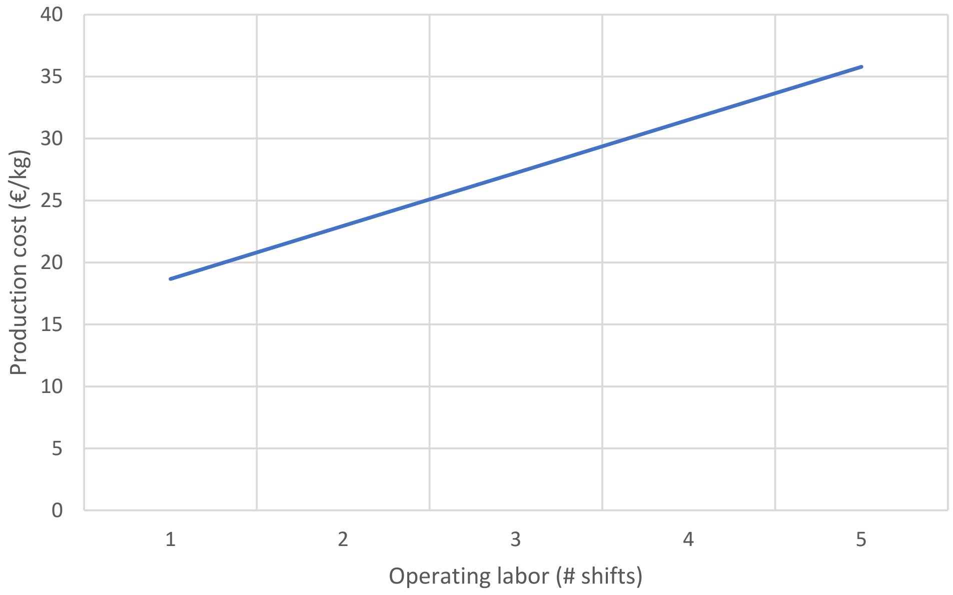

Figure S5. Sensitivity of production cost to the assumption on the amount of operating labor in the baseline scenario.

References

1. Towler, G.; Sinnott, R. *Chemical Engineering Design: Principles, Practice and Economics of Plant and Process Design*; Butterworth-Heinemann: Oxford, UK, 2012
2. Seider, W.D.; Seader, J.D.; Lewin, D.R. Reactor-separator-recycle networks. In *Product and Process Design Principles: Synthesis, Analysis and Design*, 2nd ed.; Wiley: Hoboken, NJ, USA, 2003.
3. Peters, M.; Timmerhaus, K.; West, R. *Plant Design and Economics for Chemical Engineers*, 5 ed.; McGraw-Hill Education: New York, NY, USA, 2003.
